# The efficacy of a commercial competitive exclusion product on Campylobacter colonization in broiler chickens in a 5-week pilot-scale study

**DOI:** 10.3382/ps/pew020

**Published:** 2016-03-04

**Authors:** C. Schneitz, M. Hakkinen

**Affiliations:** * Orion Corporation, P.O.Box 425, 20101 Turku, Finland; † Finnish Food Safety Authority Evira, Mustialankatu 3, 00790 Helsinki, Finland

**Keywords:** competitive exclusion, campylobacter, broiler

## Abstract

The efficacy of the commercial competitive exclusion product Broilact against *Campylobacter jejuni* was evaluated in broiler chickens in a 5-week pilot-scale study. Newly-hatched broiler chicks were brought from a commercial hatchery. After arrival 50 seeder chicks were challenged orally with approximately 10^3^ cfu of *C. jejuni*, wing marked, and placed back in a delivery box and moved to a separate room. The rest of the chicks (contact chicks) were placed in floor pens, 100 chicks per pen. Birds in two pens were treated orally on the day of hatch with the commercial competitive exclusion (CE) product Broilact, and three pens were left untreated. The following day 10 seeder chicks were introduced into the Broilact treated and untreated control pens. One pen was left both untreated and unchallenged (0-control). Each week the ceca of 10 contact chicks and one seeder chick were examined quantitatively for *Campylobacter*. The treatment prevented or significantly reduced the colonization of the challenge organism in the ceca during the two first weeks; the percentage of colonized birds being 0% after the first week and 30% after the second week in the Broilact treated groups but was 100% in the control groups the entire 5-week rearing period. During the third rearing week the proportion of *Campylobacter* positive birds started to increase in the treated pens, being 80% after the third week and 95 and 90% after the fourth and fifth rearing weeks, respectively. Similarly the average count of *Campylobacter* in the cecal contents of the Broilact treated chicks started to increase, the difference between the treated and control chicks being 1.4 logs at the end of the rearing period. Although the protective effect was temporary and occurred only during the first two weeks of the rearing period, the results of this study support the earlier observations that CE flora designed to protect chicks from *Salmonella* may also reduce *Campylobacter* colonization of broiler chickens.

## INTRODUCTION

The major cause of human bacterial enteritis worldwide is *Campylobacter*, in particular *Campylobacter jejuni*. In Europe, handling, preparation and consumption of broiler meat is estimated to account for 20% to 30% of human *Campylobacter* infections, while the estimated proportion of human cases attributed to the whole chicken reservoir via other transmission routes is up to 80% (EFSA 2011). However, highly effective interventions that reduce *Campylobacter* contamination of broiler carcasses during the slaughter process, such as chemicals and irradiation, seem not to be acceptable from the consumer's viewpoint (MacRitchie et al., 2014). Instead, effective interventions are needed at the primary production stage to reduce the load of campylobacters and cross-contamination of carcasses in the later steps of the broiler production chain (EFSA 2010).

Various approaches to reduce the *Campylobacter* colonization of poultry on farm have been suggested. These include, for example, vaccines (Buckley et al., 2010), bacteriocins (Svetoch and Stern 2010), organic acids (Jansen et al., 2014), probiotics (Ghareeb et al., 2012), and phage therapy (Kittler et al., 2013) applied to broiler chickens during the rearing period. However, these applications are still under development for *Campylobacter* and are not commercially available to be widely used in broiler industry.

Competitive exclusion (**CE**) using intestinal microbes of adult chickens for the prevention of *Salmonella* colonization of newly-hatched chicks is a well-known concept. Since early 1970s, it has been successfully used in Finland, where a commercial CE product, Broilact, is applied to the majority of broiler chicks (Hirn et al., 1992). A short-term in vivo study by Hakkinen and Schneitz (1999) suggested that the same CE product could also prevent or reduce *Campylobacter* colonization of young chickens. This study was conducted to test if the efficacy of Broilact against *Campylobacter* colonization lasts the full 5-week rearing period.

## MATERIALS AND METHODS

### Experimental Procedures

The treatment material was the commercial CE product Broilact (Orion Corporation, Espoo, Finland). It is a selected and well-characterized mixed culture derived from the cecal flora of an adult healthy hen. Broilact consists of strictly and facultative anaerobic strains reflecting the normal flora of poultry. It is entirely free from all spore-forming organisms and contains only one Gram-negative facultative anaerobic rod, a well-characterized *E. coli* strain that was chosen for its sensitivity to all tested antibiotics. The challenge organism was a *Campylobacter jejuni* strain isolated from a broiler chicken (Finnish Food Safety Authority Evira). Altogether 550 unsexed newly-hatched Ross broiler chicks were brought from a commercial hatchery. The birds were given ad libitum a wheat (41.9%), barley (20.0%), and soybean meal (30.0%) based diet which did not contain any growth-promoting antibiotics or anticoccidials. The composition of the diet simulated that of a commercial starter diet and was fed the entire rearing period. Regular tap water was available ad libitum from bell drinkers.

For enumeration of *Campylobacter* mCCD agar (Campylobacter Blood-free Selective Agar, Oxoid, Hampshire, UK) supplemented with 32 mg/L cefoperazone (Oxoid, Hampshire, UK) was used. The dilution solution was 0.1% peptone water. The plates were incubated at 41.5°C for 2 d in microaerobic conditions. After incubation, the typical flat grey colonies growing on the plates were counted. The colonies were checked to be *Campylobacter* microscopically.

The trial consisted of five pens of chicks, 100 contact chicks, and 10 seeder chicks per pen. Two pens received Broilact; two were untreated control pens and one pen served as 0-control and was left untreated and unchallenged. After arrival to the rearing facilities the chicks were randomly taken first to the control pens, and then during administration of Broilact to the test pens. However, before administering the test material, 50 seeder chicks were challenged by oral gavage with a 10^−3^ dilution of a 24-hour culture of *C. jejuni* in Brucella broth (Beckton Dickinson and Company, Sparks, MD). The dose was 4.5 × 10^3^ viable cells per bird. The size of the challenge dose was determined by quantitative plating of the culture. The seeder chicks were wing marked and kept in the delivery box in a separate room for 24 hours before introducing them to their respective pens. Five of the seeder chicks were taken to the laboratory and asphyxiated to ensure that they had started to excrete *Campylobacter*. The birds were reared on peat litter in floor pens. All pens were in the same room and different treatment groups were separated by plastic curtains and a distance of approximately 6 m. The size of a pen was 2 × 2 m (4 m^2^) and parallel pens (same treatment group) were separated only by a wire-netting fence. Chicks in the treatment groups were given by oral gavage 1 mg of Broilact in a dose volume of 0.3 mL of regeneration agent solution which was prepared according to the manufacturer's instructions.

Each week 10 contact chicks and 1 seeder chick were asphyxiated with CO_2_ from the Broilact treated and control pens and 10 birds from the 0-control pen. Their cecal contents were diluted in 0.1% peptone water and the challenge organism was determined quantitatively on mCCD agar. In addition, undiluted cecal contents of the seeder chicks were streaked on mCCD agar plates.

### Statistical Procedures

Log10-transformed values of the microbial density estimates were used throughout, except for the birds from which no challenge bacteria could be isolated (<10^2^), where the log10 value 0 was instead used. The tests applied are included in the “stats,” and the plotting routines in the “graphics” library of the software from the R Foundation for Statistical Computing (http://cran.r-project.org; R version 3.2.0). The normality of the data distribution was checked with the Shapiro-Wilk test using the shapiro.test() module. As the treated chick data, in particular, were not normally distributed but skewed to the lower microbial densities, the nonparametric Wilcoxon 2-sample test (also known as the Mann-Whitney test) was applied for comparison of the treated and control groups; the software module used was wilcox.test(). The group-specific results were plotted with the boxplot module of R graphics library. The module displays the median, a box formed by the quartiles, the range and outliers of the data in the groups. The *P*-values were calculated on the null hypothesis that there is no location shift in the data distributions of the compared groups; the alternative hypothesis was 2-sided.

## RESULTS AND DISCUSSION

**Figure 1.**
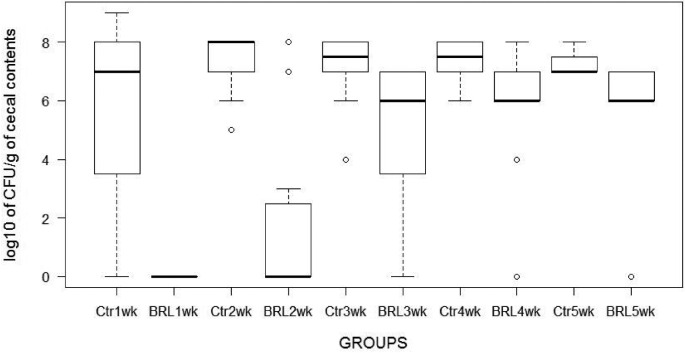
Effect of Broilact (Orion Corporation, Espoo, Finland) on the colonization of *Campylobacter jejuni* in the ceca of broiler chicks determined weekly during the 5-week rearing period. The plot shows the results of 20 Broilact treated and 20 control chicks taken each week. The heavy line indicates the median, the box extends from the lower to the upper quartile, the whiskers extend from the box to show the range of the data, and the small circles indicate outliers in the groups. Ctr = untreated Control; BRL = treated with Broilact.

The results are shown in Tables 1 and 2 and in Figure 1. The challenge organism spread fast among the control chicks and had colonized their ceca at a high level already one week after introducing the seeder chicks into the groups, the mean infection factor (**IF** = log_10_ cfu g^−1^; Mead et al., 1989; Schneitz and Hakkinen, 1998) being 5.6 when the 20 Broilact treated chicks examined were *Campylobacter* negative (Table 1). Two weeks after challenge the mean IF of the control chicks was 7.3 and remained at this level to the end of the rearing period, all 20 control chicks examined being *Campylobacter* positive. The 2-week results are similar to those of the 2-week study by Hakkinen and Schneitz (1999). The mean IF of the Broilact treated birds was 1.4 in this study and 1.8 in the previous one, which indicates that the average cecal concentration of *Campylobacter* is below 100 cfu /g. Furthermore, less than one third of the treated birds were colonized (30% and 29%, respectively) (Table 2). From the second week on, the loads of *Campylobacter* started to increase also in the Broilact treated groups. At 5 weeks-of-age, 90% of the 20 birds examined were *Campylobacter* positive the mean IF of the Broilact treated chicks being 5.9 which was 1.4 log_10_ lower than that of the control groups. The week-by-week results of the *Campylobacter* counts are presented as a box-and-whisker plot in Figure 1. Each week the counts in the treated chick groups were statistically highly significantly lower than those in the untreated control groups (*P*-values of ≪0.001).

**Table 1.** Efficacy of Broilact (Orion Corporation, Espoo, Finland) against *Campylobacter jejuni.*

	IF^1^									
	
Treatment	Week 1		Week 2		Week 3		Week 4		Week 5	
Broilact	0.0	(4.5)	1.4	(4.5)	4.8	(7.0)	5.9	(7.0)	5.9	(5.5)
Control	5.6	(7.0)	7.3	(7.0)	7.2	(8.5)	7.4	(7.0)	7.3	(7.0)
0-control	0.0	–	0.0	–	0.0	–	0.0	–	0.0	–

^1^Infection Factor (IF) is the logarithmic number of colony forming units of *Campylobacter jejuni* per gram of cecal contents (IF = log_10_cfu g^−1^). Each IF value is the geometric mean of the counts of the challenge organism per gram of cecal contents for 20 chicks (10 per treatment group). The bracketed IF value is the mean of 2 seeder chicks, 1 per treatment group.

**Table 2.** Proportion and percentage of *Campylobacter* positive chicks in Broilact (Orion Corporation, Espoo, Finland) treated, control and 0-control groups.

	Proportion and percentage of *Campylobacter* positive birds									
	
Treatment	Week 1		Week 2		Week 3		Week 4		Week 5	
Broilact	0/20	0%	6/20	30%	16/20	80%	19/20	95%	18/20	90%
Control	16/20	80%	20/20	100%	20/20	100%	20/20	100%	20/20	100%
0-control	0/10	0%	0/10	0%	0/10	0%	0/10	0%	0/10	0%

All chicks examined from the 0-control groups were *Campylobacter* negative. The five seeder chicks that were asphyxiated 24 hours after challenge were all *Campylobacter* positive. The number of the challenge organisms in the cecal contents of the seeder chicks remained at high level in the control groups, the mean IF varying from 7.0 to 8.5 during the 5-week rearing period while it varied from 4.5 to 7.0 in the Broilact treated groups (shown bracketed in Table 1). Similar decrease in the number of the challenge organisms in the cecal contents of the seeder chicks was also seen in trials where *Salmonella* was used as the challenge organism (Schneitz, 1992).

The protective effect of undefined and defined CE cultures against *Campylobacter* has been shown in several 1- and 2-week studies over years (Schoeni and Wong, 1994; Mead et al., 1996; Hakkinen and Schneitz, 1999; Stern et al., 2001; Zhang and Doyle, 2007) whereas published long-term studies are few. In a 5-week pilot-scale study, employing similar seeder-bird technique, Aho et al., (1992) combined Broilact and *Campylobacter* like bacteria (K-bacteria) isolated from the cecal homogenate of an adult chicken. At slaughter, the levels of *Campylobacter* carriage in cecal contents of treated birds were 1.5 to 2.0 log_10_ lower than those of controls, agreeing with the results of this study. In The Netherlands approximately 2.4 million broiler chickens were treated with Broilact and another 2.3 million served as untreated control group in a field experiment. The study consisted of 6 consecutive rearing periods of 6 weeks on 20 Broilact treated and 20 untreated control farms and ended up in 30% reduction in the number of *Campylobacter* positive farms (Bolder et al., 1995). It has been suggested that the consistent long-term use of CE cultures against *Salmonella* in Finnish broiler flocks has also contributed to the low incidence of *Campylobacter* in broilers (Aho and Hirn 1988). The birds were treated only once in this study and a retreatment during the rearing period might have boosted the effect of CE. The results support the earlier observations that CE flora designed to protect chicks from *Salmonella* may also reduce *Campylobacter* colonization of broiler chickens even if the intestinal ecology of these pathogens differs. However, reproductions of this study are needed to confirm the efficacy of Broilact against *Campylobacter* in long-term experiments.
